# Physical activity and exercise during pregnancy in Africa: a review of the literature

**DOI:** 10.1186/s12884-020-03439-0

**Published:** 2020-11-25

**Authors:** Uchenna Benedine Okafor, Daniel Ter Goon

**Affiliations:** 1 Department of Nursing Science, University of Fort Hare, 50 Church Street, 5201 East London, South Africa; 2 Department of Public Health, University of Fort Hare, 05 Oxford Street, East London, South Africa

**Keywords:** Physical activity, exercise, pregnancy, Africa

## Abstract

**Background:**

Pregnancy is an important phase in a woman’s life, with health status at this stage affecting both the woman and her child’s life. Global evidence suggests that many women engage in low levels of physical activity (PA) and exercise during pregnancy despite its beneficial effects. This is particularly the case in Africa.

**Methods:**

This article reviews the literature on levels of PA and exercise among pregnant women in Africa, highlighting the level of PA or exercise participation during pregnancy in Africa, including types of PA, factors affecting PA, beliefs about and benefits of prenatal activity, advice or counselling on PA during pregnancy in Africa, and PA interventions proposed to promote the uptake of prenatal PA. Electronic search databases used were Google Scholar, Science Direct, Scopus, EMBASE, ERIC, Medline, Web of Science, EBSCOhost, PubMed, BIOMED Central, and African Journal Online. The basic search terms were: ‘Physical activity’, ‘Exercise’, ‘Pregnancy’, ‘Pregnant women’ and ‘Africa’. A total of 40 references were found. On the basis of an analysis of titles, abstracts and the language of publication (other than English), 11 articles were rejected, and 29 articles were fully read, although two had to be rejected due to a lack of access to the full version. Finally, 27 references were included in the review.

**Results:**

Few studies exist on PA during pregnancy in Africa. The limited data available suggests that, compared to the Western world, pregnant women in Africa do not adhere to the recommendations for PA during pregnancy. Levels of participation in PA during pregnancy are low and decline as the pregnancy progresses. The majority of the studies used direct, objective measures to assess PA during pregnancy. Personal and environmental factors such as lack of time, lack of knowledge, inadequate information from healthcare providers, feelings of tiredness and an absence of social support constituted the main barriers to PA during pregnancy. The types of PA participation among pregnant women varied across studies and geographical settings.

**Conclusions:**

While published data is limited, it seems clear that the participation of pregnant women in PA during pregnancy in Africa is low and declines with advancing pregnancy. There is a need for more studies to examine the dynamics of PA during pregnancy in Africa to guide contextual interventions to improve and promote maternal health on the continent.

## Background

Physical activity (PA) as a modifiable health risk factor has been shown to contribute to the maternal health of women and their offspring. Several studies have reported the benefits of PA and exercise during pregnancy, which include reduced risk of excessive gestational weight gain [1–5], decreased risk of gestational diabetes [1, 6–9], and reduced risk of preeclampsia [10–14]. Evidence also indicates that PA during pregnancy lowers rates of preterm births [15, 16], improves sleep [17, 18], reduces the risk of caesarean section and postpartum recovery time [19–21], and reduces length of labour and delivery complications [22]. In addition, PA during pregnancy reduces fatigue, stress, anxiety and depression [10–12, 22–26], reduced lower back pain [10–12, 27], and improves wellbeing [28]. Moreover, it is known to increase heart rate, cardiac output, ventilation and energy expenditure [29, 30], reduce the risk of injury for both mother and baby [3], and improves breastfeeding outcomes [31].

Earlier studies have alluded to the benefits of physical activity for the improvement of maternal health of the mother and the baby. Evidence has shown maternal obesity is a precursor for the development of adverse maternal health outcomes (cardiovascular diseases, metabolic syndromes, and obesity) [32, 33], and the children [34, 35]. Specifically, studies have reported increased risk of fetal death [36, 37], congenital malformations such as neural tube defects [38, 39], macrosomia [40–42], and large-for-gestational-age [36, 43]. Children of obese mothers have increased perinatal complications, which include shoulder dystocia, birth injuries, perinatal asphyxia [38], and hypoglycemia or respiratory distress [38]. In addition, women with excessive gestational weight gain have higher propensity for postpartum weight retention (PPWR) [33]. Therefore, it is important to prevent excessive PPWR during the pregnancy-postpartum period [44]. Previous studies have shown antenatal exercises improves the aerobic fitness of pregnant women [13, 45, 46], and significantly lowers postpartum weight retention [47]. Viewed in this context, obese pregnant women should be provided with adequate education and support concerning prenatal exercise to improve their health outcomes and the baby; and with these manifold benefits, it is recommended that pregnant women, without complications should engage in moderate-to-vigorous PA for at least 150 minutes per week [6].

Notwithstanding the substantial evidence on the benefits of PA during pregnancy, studies have reported considerable declines in physical activity among pregnant women [48–54], with varying degrees of participation shaped by context-specific factors. Pregnant women do not meet the American College of Obstetricians and Gynaecologists (ACOG) recommendation of regular PA during pregnancy for women who are pregnant and healthy to perform 30 minutes or more of light to moderate exercise a day on most, if not all, days of the week [18].

Various factors have been shown to influence PA participation among pregnant women, including low maternal education [55–57], unemployment [58, 59], pregnancy symptoms/discomforts [3, 55, 60–62], multiparity [63], lack of strength or fatigue [3], lack of time [3, 55, 60], lack of motivation [56, 64, 65], and safety concerns or fear [62, 66]. Some studies have reported cultural and religious beliefs [66], lack of social support [61, 67] and other responsibilities [61, 68]. In addition, studies reporting environmental barriers to PA during pregnancy cited lack of access to facilities/resources [67, 69], and bad weather conditions [3, 61, 67]. An empirical understanding of the context-specific factors affecting PA participation during pregnancy in Africa is crucial to inform interventional strategies. This kind of data is rare in Africa.

While there are several studies to guide interventions and the promotion of physical activity and exercise among pregnant women in the USA [3, 25, 65, 70, 71], Canada [63, 72, 73], the United Kingdom [74–76] and other countries around the world [26, 53, 57, 61, 62, 68, 77–83], which indicate varying degrees of PA participation, and suggest the reasons for both participation and non-participation during pregnancy, scant information exist in Africa. A previous review by Mukona et al. [84] identified only two studies conducted in South Africa [56, 85], two studies in Nigeria [86, 87], and one study in Ethiopia [88]. This earlier review reported a low level of physical activity during pregnancy, largely because of lack of knowledge about prenatal activities, lack of facilities at community level and lack of time [86], thus stressing the need for the promotion of physical activity in Africa. The major form of physical activity performed by pregnant women was household activities, and physical activity declined as the pregnancy progressed [86]. In Africa, pregnancy is generally considered a time of confinement and withdrawal, a deeply held belief. While this has traditionally been the case, and is still the case in rural areas, urbanisation has brought about a decline in levels of physical activity, to the detriment of overall country health profiles, and specifically the health profiles of pregnant women. Exercise and general activities generally decrease over the course of the pregnancy, a factor associated with tiredness [85].

The value of PA and exercise in relation to the maternal health of the mother and child should be explored and promoted beyond the matter of merely reducing the direct causes of maternal morbidity; the issue should be seen as a modifiable health risk factor, as is the case with diet [55]. Given the uniqueness of pregnancy in the life cycle of a woman, health behaviours and disease risk factors are important indicators to consider during pregnancy. Research interest in health during pregnancy ought to be high, since pregnancy is a time in a woman’s life which can have lasting effects on her future health. A synthesis of the factors that influence physical activity and exercise during pregnancy in Africa may provide evidence to inform interventions on PA promotion and implementation in antenatal healthcare, which will of course be influenced by context. Despite the clear guidelines and recommendations set by various bodies and institutions, achieving sufficient levels of PA and exercise remains a global challenge, not only to the general population in Africa, but specifically to the population group of pregnant women. To our knowledge, a comprehensive review of PA during pregnancy in Africa does not exist. This knowledge gap is worrisome because the correlates, perceived benefits and barriers to PA participation among pregnant women in Africa may differ considerably from one country to another. Such a review would increase our knowledge of the context-specific issues regarding PA during pregnancy in Africa. This knowledge would help health professionals and policymakers to develop environmental and/or behavioural modification strategies and effective interventions.

Physical activity (occupational, sports, conditioning, household or other activities) is defined as any body movement produced by skeletal muscles that results in energy expenditure [89, 90]. Exercise, on the other hand, is a subcategory of physical activity, and is a planned, structured, repetitive activity to improve or maintain physical fitness [89, 90]. In this review, PA and exercise will be used interchangeably to mean the same thing, as the pregnant woman engages in both for the improvement of maternal outcomes. The purpose of this study was to conduct a narrative literature review on the level of PA or exercise participation during pregnancy in Africa, including types of PA, factors affecting PA, beliefs about and benefits of prenatal activity, advice or counselling on PA during pregnancy in Africa, and lastly, PA interventions proposed to promote the uptake of prenatal PA.

## Methods

### Search strategy

Our electronic search crossed six databases: Google Scholar, Science Direct, Scopus, EMBASE, ERIC, Medline, Web of Science, EBSCOhost, PubMed, BIOMED Central and African Journal Online databases. These were consulted for any published review articles or original research articles, regardless of year of publication that yielded information on the levels and correlates of PA participation during pregnancy, including the beliefs, perceived benefits, barriers and attitudes of pregnant women concerning PA and exercise participation during pregnancy in Africa. In addition, we extended the search for articles so as to include references of the identified publications in this narrative review.

The search terms used were: ‘Physical activity’, ‘Exercise’, ‘Pregnancy’, ‘Pregnant women’ and ‘Africa’.

The search excluded research articles published in languages other than English and whose full texts were not accessible.

**Fig. 1:**
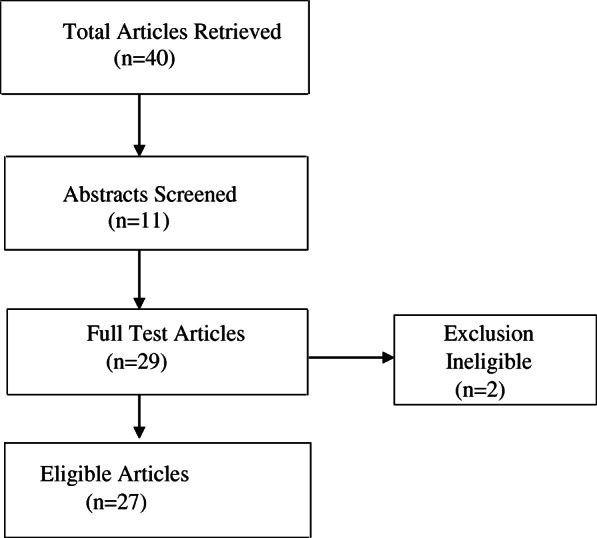
Flow diagram of articles searched

A total of 40 original articles and review articles on physical activity during pregnancy in Africa were included in this narrative review. Of these, 27 studies were finally considered for the analysis [55, 56, 58, 59, 86–88, 91–110]. Figure 1 presents a flow diagram of the procedure that resulted in the final list of articles considered for analyses.

## Results

Of the 40 articles retrieved, 27 studies met the inclusion criteria for the narrative review (Fig. 1). Seven studies utilised direct or objective measures to assess PA during pregnancy (accelerometers, pedometers, combined heart rate and accelerometer device, and Borge Rating of Perceived Exertion (RPE) [88, 92–95, 103, 107], and 16 studies employed non-objective or indirect measures [56, 58, 59, 86, 87, 97, 102, 104, 106, 108, 109]. Four studies include the Pregnancy Physical Activity Questionnaire (PPAQ) [58, 86, 100, 101], 11 applied surveys or questionnaires (Global Physical Activity Questionnaire (GPAQ), International Physical Activity Questionnaires, or interviews (Table 1). Notably, the PPAQ is a validated tool for evaluating PA among pregnant women.

**Table 1:** Included studies for literature review

Country/Author(s)	Settings	Sample and sampling technique	Trimester or gestational period	Design	Measure of PA	Main Findings
South Africa [55]	Soweto (Urban)	13 purposively selected participants aged 19–41 years	3rd (29–33 weeks)	Qualitative	Indirect measure: Semi-structured interviews	• Demonstrated positive beliefs about PA, but participants remained inactive during the prenatal period. • PA limitations included lack of time, lack of finances and inadequate information from healthcare providers. • There was also an absence of social support network to facilitate PA participation. • Findings suggested a holistic approach to improve PA compliance during pregnancy, inclusive of PA education and exercise opportunities within a community setting.
South Africa [56]	Vanguard, Western Cape (Urban)	34 stratified randomly selected participants, aged 17–36 years	All	Qualitative exploratory study	Indirect measure: Interview	• Pregnant women showed an interest in participating in PA during pregnancy and requested more information and a possible intervention programme. • Participants were aware of the benefits of being physically active during pregnancy, but knowledge levels varied. • Participants were unsure of the types of PA recommended for safe practice in pregnancy. • Participants said that they did not receive PA-related advice and information from health care providers. • Participants expressed interest in PA clubs or antenatal PA classes for accessibility to safe and regular PA.
Ethiopia [58]	Mekelle	299 (16–36 years)	All	Cross-sectional	Indirect measure: PPAQ	• Only 8.4% women met the international recommended guideline for PA during pregnancy. • Age group of 26–35 years (AOR: 2.69, 95% CI: 1.07–6.78), attending non-formal education (AOR: 13.50, 95% CI: 2.65–68.91), and unemployment (AOR: 5.23, 95% CI: 1.34–20.38) were significantly associated with a higher risk of sedentary activity status. • Being married (AOR: 0.26, 95% CI: 0.09–0.73), having two children (AOR: 0.13, 95% CI: 0.03–0.59), and traveling an hour or more to health facilities (AOR: 0.31, 95% CI: 0.11–0.89) were significant positive predictors of physical activity participation. • Majority (70.6%) received advice about PA from health professionals • Most common reported reason for not exercising during pregnancy was fear of miscarriage. • Walking was the most commonly reported mode of exercise (86.3%).
Ethiopia [59]	Tigray	442 (18–38 years)	Not stated	Cross-sectional	Self-administered questionnaire	• Majority (78.1%) of the women were physically active • Generally, expended less total energy during pregnancy 141.23 MET-h/week • Expended highest amount of energy (69.4 MET-h/weeks) on household activities • Primiparous women were 7.68 times more likely to be inactive as compared to multiparous women • Education level of mothers had significant association with women’s level of physical activity during pregnancy • Women with history of miscarriage had 8.05 times higher odds of becoming physically inactive during pregnancy as compared to those without history of miscarriage [AOR = 8.045; 95% CI (3.325, 19.465)).
Nigeria [86]	Ibadan (Urban)	453 conveniently selected participants (mean age: 30.89 ± 4.44 years)	2nd & 3rd	Cross-sectional	Indirect measure: PPAQ	• Half the participants were sedentary. • Most of the energy expended by the pregnant women was on household chores. • Number of children, stage of pregnancy, gravidity and employment status had a significant influence on the physical activity levels of the pregnant women. • Increased number of children and gravidity, and advanced stage of pregnancy, significantly predicted increased likelihood of being sedentary.
Nigeria [87]	Ile-Ife (Urban)	189 consecutively selected (mean age: 28.9 ± 4.63 years)	All	Cross-sectional	Indirect measure: Self-administered questionnaire	• Most women had knowledge of pelvic floor muscle strengthening, back care, relaxation and breathing exercises from antenatal exercises. • Swimming and cycling were not known as antenatal exercises. • Most women affirmed antenatal exercises reduced back pain, promoted ability to cope with labour and delivery, and prevented excessive weight gain. • Knowledge about the benefits of antenatal exercises was not influenced by maternal sociodemographic characteristics, but age was found to significantly influence knowledge about contraindications to antenatal exercises. • Knowledge of exercise during pregnancy was influenced mostly by tiredness, lack of will to exercise, and insufficient information on exercise. • The majority of pregnant women demonstrated inadequate knowledge about antenatal exercises. However, the women had positive attitudes towards exercise.
Ethiopia [88]	Jimma (Urban)	304 conveniently selected participants aged 20–27 years	All	Cross-sectional	Direct measure: Combined Uniaxial Accelerometer and Heart Rate Sensor	• Overall level of PA was low-AEE (kJ/kg/day) and PAL • Most women spent most of their time in sedentary and light intensity activities, with small amounts of moderate to vigorous activity. • Gestational age and degree of adiposity were both independently associated with lower activity and fitness levels, while muscle mass was independently associated with higher activity and muscular fitness levels.
Burundi [91]	Rural/Urban	150 conveniently selected participants aged 18–40 years	All	Quantitative	Indirect measure: Self-administered questionnaire	• Most participants adopted sedentary lifestyles during pregnancy (88.0%), and had a negative view of exercise and PA during pregnancy (84.6%).
Egypt [92]	Kafrelsheikh (Urban)	20 healthy pregnant women aged 20–25 years	2nd & 3rd (20–36 weeks’ gestation)	Randomised, controlled trial (RCT)	Direct measure: Pelvic floor muscle (PFM) using Peritron® to measure vaginal squeeze pressure	• Significant differences between both groups in mean PFM at 36 weeks gestation (WG); and a significant correlation between PFM strength at 36 WG and mode of delivery (vaginal delivery; r = 0.58, p < 0.05; caesarean delivery; r = -0.49, p < 0.05). • Recommended pelvic floor muscle exercises for health during pregnancy as a safe and inexpensive strategy for increasing the vaginal delivery rate.
Egypt [93]	Cairo (Urban)	100 (50 control group; 50 intervention group). Age: 20–35 years	Not stated	Prospective, interventional and controlled	Direct measure: Supervised exercises (aerobic, stationary cycling, treadmill walking, stretching, pelvic curls, tailor press back bridge, crunches)	• Exercise group exhibited significant improvement in depressive symptoms after the aerobic exercise programme compared to baseline (p < 0.001), while the control group demonstrated no significant change over time. • Supervised exercise during pregnancy has a positive effect on antenatal depression as a primary and secondary preventive strategy.
Egypt [94]	Aswan (Urban)	360 (Mean age: 25.± 2.4 years)	10–39 weeks gestation	Prospective Cohort	Direct measure: Supervised regular walking 5 times per week for 30 minutes	• Regular walking significantly reduced the occurrence of preeclampsia (OR = 0.120, 95% CI; 0.015–0.970; p = 0.037), postdate pregnancy (OR = 0.274; 95% CI = 0.099–0.759; p = 0.008), excessive weight gain (OR = 0.220; 95% CI = 0.114–0.424; p = 0.000) and caesarean delivery (OR = 0.519; 95% CI = 0.316–0.841; p = 0.007). • Only a small proportion of pregnant women engaged in moderate to high (1.2%) PA during pregnancy.
Egypt [95]	Urban	60 participants, randomly assigned to 30 in the control group and 30 in the intervention group	2nd and 3rd (≥ 14 weeks gestation)	Clinical control trial	Direct measure: Supervised antenatal exercises	• Exercises had no effect on the mode of delivery and maternal activity during gestation in both groups. • Antenatal exercise is very effective in decreasing adverse effects in older primigravida and their offspring.
Egypt [96]	Cairo (Urban)	40 (Age: 25–35 years)	2nd (20–24 weeks)	Prospective, interventional and controlled	Direct measure: Walking on treadmill	• Moderate intensity aerobic exercises were effective in reducing fasting blood glucose and fasting insulin levels in pregnant women at risk of gestational diabetes mellitus in both interventional and control groups.
Egypt [97]	Urban	64		Quasi-experimental prospective study	Indirect measures: Structured interviewing, questionnaire	• Stretching and physical exercise in women with mild preeclampsia promoted positive foetomaternal outcomes and did not pose greater maternal or neonatal risks than among those who did not practice.
Kenya [98]	Kakamega County	306 conveniently selected participants aged 15–40 years	All	Descriptive survey	Indirect measure: Self-administered questionnaire	• About 17% did not know that exercise is useful in pregnancy. • Participants indicated exercise prevented incontinence (80.4%), decreased risk of preeclampsia (71.6%), and decreased risk of gestational diabetes (65.7%) and hypertension (68.6%) • Strong association between the level of education and knowledge on the role of exercise during pregnancy (X^2^ = df; 3 = 39.109;p = 0.02)
Kenya [99]	Rongo (Rural)	100 (Age: ≤19–45 ≥ years)	2nd & 3rd	Longitudinal	Indirect measure: International Physical Activity Questionnaire	• Women dedicated 78% of their time to physical work and only 22% to leisure activities per day. • On average, women were active in their 2nd trimester as well as their 3rd trimester of pregnancy. • Habitual PA of pregnant women in the setting included domestic, productive and leisure activities. • Daily energy expenditure was relatively high
Nigeria [100]	Maiduguri (Urban)	398 randomly selected participants 18->30 years	All	Analytic cross-sectional survey	Indirect measure: Pregnancy Physical Activity Questionnaire (PPAQ)	• Most (86.4%) pregnant women did not participate in PA, and only 14.6% achieved the recommended levels of PA. • PA significantly decreased from 1st trimesters to 2nd and 3rd trimesters. • Sport/exercise was associated with enhanced physical health and health-related quality of life (HQoL) (r = 0.142,p < 0.01). • Pregnant women with sufficient PA were four times more likely to report a high quality of life, physically (OR: 4.33, 95%, CI: 1.36–13.80) • The study recommended sports/exercise as an important aspect of PA to prevent delivery interventions and improve the physical wellbeing of pregnant women, at least in that setting.
Nigeria [101]	Osun State, south west (Urban)	289 purposively selected participants (mean age: 29.8 ± 5.11 years)	All	Cross-sectional	Indirect measure: PPAQ	• Most participants were involved in light intensity and household PA (1263.6 ± 633.4) and low levels of vigorous intensity PA (6.4 ± 6.8) • Both the mean of participation and the occupational PA were highest in the 2nd trimester. • The 3rd trimester had the highest mean fatigue score. • There was a significant relationship between pregnancy-related fatigue and physical activity.
Nigeria [102]	Enugu (Urban)	350 purposively selected participants	All	Longitudinal cohort	Indirect measure: Self-administered questionnaire	• The majority (82.9%) of the participants practised antenatal exercise, particularly aerobic exercises (76.2%). • The majority exercised less than five days a week (70.0%) and ≥ 30 minutes daily (63.4%). • Most exercised based on self-prescription (39.0%). • Antenatal exercise practice and patterns did not improve postpartum health-related quality of life. • The study recommended improved education and supervision of antenatal exercise for better postpartum health outcomes.
Nigeria [103]	Owerri (Urban)	70 (simple random assignment into exercise and control groups)	2nd (20 weeks gestation)	Randomised, controlled trial	Direct measure: Borge Rating of Perceived Exertion (RPE)	• Exercise in pregnancy significantly lengthened period of gestation. • Exercising women were more likely to carry their pregnancies to full term than those who did not exercise. • Exercise could serve as a means of preventing preterm births.
Nigeria [104]	Urban/Rural	361		Cross-sectional	Indirect measure: Questionnaire	• Pregnant and nursing women demonstrated high engagement in physical exercise, which was undertaken by self-prescription. • Higher education is a significant determinant of exercise participation during pregnancy in Nigeria.
Nigeria [105]	Urban	30 (n = 16 control group; n = 14 intervention group) randomly selected participants aged 18–45 years	Not stated	Intervention study	Direct measure: Supervised aerobic exercises	• The study showed that aerobic exercises combined with education on sleep hygiene significantly reduced levels of insomnia and fatigue in pregnant women, which was not possible with the use of education only.
South Africa [106]	Soweto (Urban)	332 (Mean age: 29.5 ± 5.8 years)	2nd & 3rd (14–33 weeks gestation)	Longitudinal cohort	Indirect Measure: Global Physical Activity Questionnaire (GPAQ)	• Half of the women were classified as being active in the 2nd trimester, however, significantly fewer women participated in PA in the 3rd trimester • Total PA decreased significantly as the pregnancy progressed. • Walking for transport constitutes 80% of the total MVPA. • The study showed that pregnant women spent an average of five hours per day sitting.
South Africa [107]	Soweto (Urban)	210 (Mean age: 30.4 ± 5.8 years)	2nd & 3rd (14–33 weeks)	Observational, longitudinal study	Direct measure: Hip-worn triaxial accelerometer (ActiGraph GT3X+, ActiGraph, Pensacola, FL)	• Significant decline in PA from 2nd to 3rd trimester (12.8 ± 4.1 mg vs. 9.7 ± 3.6 mg, p < 0.01) with a high prevalence of overweight/obesity and HIV. • PA at 29–33 weeks and changes in PA were inversely associated with weight change at 29–33 weeks (β = 0.24; 95% CI-0.49; -0.00; p = 0.05 and β = -0.36; 95% CI-0.62; -0.10; p = 0.01, respectively) • No significant associations between PA and birth outcomes.
South Africa [108]	Vhembe, Ngovhela (Rural)	59 randomly selected participants aged < 18->30 years	2nd & 3rd	Cross-sectional	Indirect measure: Self-administered questionnaire	• Women demonstrated an average knowledge of types of antenatal exercises, their benefits and contraindications. • Women had no knowledge of pelvic floor exercises. • Antenatal exercise participation was low.
Zambia [109]	Lusaka (Urban)	300 (Mean age: 29.4 years)	All	Cross-sectional survey	Indirect measure: Self-administered questionnaire	• Exercise practice was significantly associated with level of education. • Most of the participants exhibited inadequate levels of knowledge on ideal exercises during pregnancy. • Most reported a lack of knowledge on how to perform antenatal exercises, feelings of fatigue and general discomfort as barriers to exercises participation. • Walking was identified as the most common type of exercise performed by the women. • Participants did not know any specific antenatal exercises; consequently, they were not able to practise correct exercises during pregnancy.
Ethiopia [110]	Butajira	247 (15–45 years)	3rd (31–34 weeks	Community-based prospective cohort study	Indirect measure: Global Physical Activity Questionnaire	• About 47.2% women engaged in vigorous physical activities • Low birthweight at term was significantly associated with vigorous physical activity (AOR = 2.48; CI: 1.01–6.09), prolonged standing (AOR = 3.37; CI: 1.14–9.93), and squatting (AOR = 2.61; CI: 1.04–6.54)

*RCT *Randomised Control Trial; *PPAQ *Pregnancy Physical Activity Questionnaire

### Characteristics of included studies

Characteristics of the 27 included studies are displayed in Table 1. Four were prospective cohort studies [92, 94, 97, 105], thirteen were cross-sectional studies [58, 59, 86–88, 91, 98, 100, 101, 104, 108–110], four were longitudinal studies [99, 102, 106, 107], four were randomised controlled trials [92, 95, 96, 103] and two were qualitative exploratory study [55, 56].

Most studies were carried out in Nigeria [86, 87, 100, 102–104], six were conducted in Egypt [92–97], five in South Africa [55, 56, 106–108], two in Kenya [98, 99], four in Ethiopia [58, 59, 88, 110], one each in Burundi [91], and Zambia [109]. The majority of the studies were conducted in urban regions [55, 56, 58, 86–88, 92–97, 100–103, 105–107, 109], three were in rural areas [98, 99, 108], and two were conducted in a combination of urban/rural settings [91, 101, 110].

### Trimester or gestational period

Ten studies focused on the second trimester of pregnancy [86, 88, 92, 94–96, 99, 103, 107, 108] nine studies assessed women in the third trimester of pregnancy (87,89,93,95,96,98,100,108,109] and nine studies (57,89,92,99,101,102,103,107,110] included all the trimesters or the entire gestational period. Three studies did not state the gestational stage [59, 93, 105].

Level of physical activity participation during pregnancy.

Some of the studies across all trimesters reported declines in PA activity during pregnancy as the pregnancy progressed [59, 99–101, 106, 107]. Regardless of the gestational stage, levels of PA participation were generally low [86, 88, 91, 100, 101, 108].

### Types of physical activity participation

The types of PA participation among pregnant women varied across studies and different geographical settings. Most studies reported that pregnant women engaged primarily in sedentary activities (sitting, household chores) [59, 86, 88, 99, 101, 106], walking [94, 106, 109], jogging, aerobics [102], floor exercises, Two studies reported pregnant women had little knowledge concerning the types and benefits of PA participation during pregnancy [98, 101, 109], but at the same time exhibited positive attitudes towards exercise [101].

### Factors affecting physical activity participation during pregnancy

Several factors influencing PA during pregnancy are summarised in the review (Table 1). Studies cited lack of time [55], lack of finances [55], lack of knowledge and inadequate information from healthcare providers [55, 56, 59, 109], feelings of tiredness [109] and the absence of social support [55] as factors affecting PA participation during pregnancy. One study found that number of children, stage of pregnancy, gravidity and employment status had a significant influence on levels of physical activity during pregnancy [6].

### Beliefs about and benefits of physical activity during pregnancy

Regarding beliefs about and benefits of PA participation, two studies reported that participants had positive beliefs about PA and exercise during pregnancy [55, 88] and two studies reported that participants had negative perceptions of PA and exercise during pregnancy [91, 98, 108]. Three studies reported that exercise prevents incontinence, decreases risk of preeclampsia [94], decreases risk of gestational diabetes [92, 98, 103], hypertension [107] and excessive weight gain [87, 94], decreases the risk of caesarean delivery [87, 94], prevents preterm births [103] and improves depressive symptoms [93]. Two studies found no significant association between PA and birth outcomes [95, 107].

### Advice or counselling from health professionals on physical activity participation during pregnancy

Two studies reported that the information provided by health professionals on PA during pregnancy was inadequate [55, 56]. One study stated that pregnant women exercised based on self-prescription [107].

### Physical activity interventions

Some studies highlight intervention measures for promoting PA during pregnancy. One study recommended pelvic floor muscle exercises during pregnancy as a safe and inexpensive strategy for increasing the vaginal delivery rate [92], two studies recommended the provision of education and supervision during antenatal exercises for better postpartum health outcomes [55, 93, 102], and one study recommended the provision of PA and exercise opportunities in the community setting [55].

## Discussion

In this narrative literature review, we determined the level of PA or exercise participation during pregnancy in Africa, including types of PA, factors affecting PA, beliefs about and benefits of prenatal activity, advice or counselling on PA during pregnancy in Africa, and lastly, PA interventions proposed to promote the uptake of prenatal PA. We found low and patchy levels of knowledge regarding the benefits and types of antenatal exercise that are recommended for pregnant women. Most pregnant women engaged primarily in sedentary activities (sitting, household chores), had little knowledge concerning the types and benefits of PA participation during pregnancy, but at the same time exhibited positive attitudes towards exercise. In addition, lack of time, lack of finances, lack of knowledge and inadequate information from healthcare providers, feelings of tiredness and the absence of social support were factors affecting PA participation during pregnancy. Pregnant women have mixed beliefs about PA participation and its benefits and the information provided by health professionals on PA during pregnancy was inadequate. Intervention measures for promoting PA during pregnancy included the provision of education and supervision during antenatal exercises for better postpartum health outcomes, and provision of PA and exercise opportunities in the community setting. Overall, our findings resonate with findings in other studies on prenatal PA showing low participation in PA during pregnancy, a decline in PA as the pregnancy progresses, amidst lack of PA advice from health providers; it also adds to calls to initiate interventions to encourage and promote PA during pregnancy in the antenatal health care continuum. The findings from this review highlight the need to design interventions that would address the reasons for inactivity during pregnancy. The onus relies on the healthcare providers and policy makers to integrate prenatal activity as part of the antenatal health care service. Pregnant women ought to be educated on the importance of PA for maternal and fetal health; they also need support and motivation to engage in PA during pregnancy. Inter-collaborative support of health providers, partner, family members, friends and organisations is desirable to promote physical activity in the community.

Most of the studies reported low and decreasing levels of PA during pregnancy in Africa [86, 88, 91, 94, 100, 101, 106–108]. This finding is consistent with other studies conducted in different countries, using varying methodological designs and methods for assessing PA, which also observed that few women exercise or participate in PA during pregnancy [48, 52–54, 111]. Reasons for and against participation in PA and exercise varied across countries and was shaped by context-specific factors. Muzigaba et al.’s [56] study on the perceived role and influencers of physical activity among pregnant women from low socioeconomic parts of urban communities reported that 44% of pregnant women in South Africa were physically inactive during pregnancy. Notably, in most developing countries, including Africa, the wave of epidemiological and nutritional transition has changed the lifestyle and the behaviour of people drastically, due to both modernisation and urbanisation, thereby promoting physical inactivity and associated health risks [112]. Understanding the factors influencing PA participation during pregnancy in the African context is important for the development of effective maternal health promotion strategies.

Across studies, types of PA varied across geographical settings. Our review indicate that most pregnant women in Africa participated in light-intensity and household activities [59, 86, 88, 99, 101, 106]. Other studies conducted outside Africa have reported household and occupational activities as the most predominant prenatal activities in pregnancy in China [48, 82], Taiwan [113], Portugal [114], Serbia [57], and Brazil [53]. Anecdotally, in innumerable communities in Africa, pregnant women work extraordinarily long hours in a variety of physically demanding tasks. The predominant means of economic survival, particularly in rural African settings, is subsistence farming and petty trading. Besides this, pregnant women work on farms, care for domestic animals and perform numerous household chores such as caring for children and elders, fetching water, collecting firewood, preparing meals and washing clothes. However, these activities alone are insufficient to produce the desired health benefits. Worryingly, some cultures prevent pregnant women from engaging in any form of physical activity because of cultural or religious beliefs. Given the indisputably positive effects of PA and exercise on maternal health outcomes, there is a need to provide health education or awareness on the importance of PA during pregnancy among reproductive-aged women in order to improve the health of the mother and child. Research to explore the cultural or religious imperatives of PA or exercise during pregnancy in some African cultures is imperative. Currently, few such studies exist.

This narrative review study highlighted the barriers to PA participation among pregnant women in Africa. From an African perspective, an in-depth understanding of the underlying factors preventing wholehearted PA participation among pregnant women might yield important insights about approaches and areas of emphasis in intervention programmes. Such an understanding might go a long way toward improving participation, and might suggest feasible options for the promotion of sustained physical activity and exercise during pregnancy. The findings of this review demonstrate that pregnant women in Africa, primarily do not engage in physical activity because of a lack of time [55], and a lack of knowledge and inadequate information from the healthcare providers [55, 56, 59, 109]. Other studies have reported similar findings elsewhere, indicating a lack of time [60, 115–117], and inadequate knowledge about physical activity [10, 118, 119]. In addition, age, the number of children, stage of pregnancy, and lower level of education, and employment status significantly influenced the level of physical activity during pregnancy in Africa [59, 85, 86, 98, 104, 109]. This proved consistent with other studies that reported age [120], low education [53, 57, 121], pregnancy trimester [122, 123], and employment [121] as correlates affecting physical activity during pregnancy. Women who attain higher level of education are more knowledgeable about PA during pregnancy [48, 59]. Interventions to educate women with lower education are imperative to encourage women to participate in prenatal activity.

Consistent with studies conducted elsewhere, this review found that low maternal knowledge of the benefits of PA during pregnancy [59, 124, 125], pregnancy symptoms/discomforts [3, 61, 62], multiparity [63], fatigue or lack of strength [3, 61], lack of time [3, 61, 67], lack of motivation and self-confidence [64, 65, 67] and fears or safety concerns [62, 66] all hinder PA participation during pregnancy. Interpersonal (social) barriers are cultural and religious beliefs [66], lack of social support [61, 67], and other responsibilities [61, 68]. Some studies have reported environmental barriers to PA during pregnancy, citing lack of access to facilities or resources [67, 69] and bad weather conditions [3, 61, 67]. Clearly, lack of information, motivation, and support for PA or exercise during pregnancy are general barriers, which may be addressed by providing regular advice and counselling on safety, benefits, and types of PA recommended during pregnancy. Health professionals should undertake this as part of antenatal or maternity health care. Understanding other country and regional-specific factors affecting PA during pregnancy is crucial, since contextual knowledge forms a component of quality antenatal and obstetric healthcare services to this special population in Africa. Africa-based information of this kind is scant.

The finding of this review study demonstrated contrasting beliefs about PA and exercise during pregnancy. Whilst two studies indicated pregnant women had positive beliefs about PA and exercise during pregnancy [55, 87], three studies reported opposite perceptions about physical activity and exercise during pregnancy [91, 98, 108]. Beliefs are subjective in nature and vary between cultures. Providing women with the relevant prenatal physical activity advice and recommendations may change their negative perceptions about physical activity and possibly propel them toward engaging in physical activity during pregnancy. Advocacy on the benefits of physical activity during pregnancy is warranted. Interestingly, some of the studies reported women affirming the benefits of physical activity. The majority of studies cited participation in physical activity as helping to decrease the risk of gestational diabetes [92, 98, 103], excessive weight gain [87, 94], and also decreasing the risk of caesarean delivery [87, 94]. The regular prenatal physical activity has physical and psychological health benefits to the pregnant mother. Elsewhere, studies have shown that physically active pregnant women are less likely to exhibit excessive gestational weight gain [3–5], are at reduced risk of gestational diabetes mellitus [1, 6–9], and of developing preeclampsia [10–14]; the forementioned being factors that are likely to have long-term adverse effects on the mother and the child [126, 127]. Other health benefits include reduction of fatigue, stress, anxiety and depression [10–12, 22–26], and reduced lower back pain [10–12, 27]. Despite these health benefits accruable from being physically active during pregnancy, prenatal physical activity of women, in Africa, is reportedly low. Viewed from the clinical and health standpoint, it is important to encourage pregnant women in Africa to participate in PA to promote maternal health outcomes.

Our review indicates that inadequate information from health professions on PA during pregnancy [55, 56] is a factor preventing participation in PA. Also, pregnant women tended to exercise based on self-prescription [102]. Given the beneficial effects of PA on pregnancy outcomes, there is need to encourage and provide proper, safe and sustainable guidelines on PA during pregnancy. Providing information about the advantages of PA during pregnancy and making women aware of existing PA guidelines is important to give women a good understanding of their condition and inform their decision-making. Healthcare providers are important sources of support for PA during pregnancy [67, 128]. They have a crucial role, which includes clarifying the benefits and emphasising the importance of PA during pregnancy [10]. However, as reported in other studies, healthcare providers in Africa provide little or no information regarding PA during pregnancy [116, 129–131]. Watson et al. [55] conducted a study involving urban South African pregnant women, finding that health professionals seldom provide advice or counselling on PA or exercise during pregnancy. Some pregnant women and obstetric care providers are uncertain whether prenatal PA increases the risk of miscarriage, or causes growth restriction, preterm birth, fatigue or harm to the foetus, an uncertainty which constitutes a barrier to being active [132]. Due to this lack of information from professionals, many pregnant women turn to family, friends or the media for advice regarding pregnancy. Healthcare providers are the gatekeepers of accurate information among communities, and can have a strong influence on healthy and safe physical activity levels among pregnant women. The growing rise of non-communicable diseases in Africa warrants the prioritisation of PA counselling in antenatal clinics. Therefore, health professionals working in maternity units should integrate physical activity counselling in the antenatal routine healthcare visit of women. Providing awareness on the importance of PA during pregnancy would undoubtedly facilitate behavioural change. Empirical evidence on sources of information for PA during pregnancy in the context of pregnant women in Africa, where scant data exists, is important to guide PA healthcare interventions.

Some studies in this review recommended the provision of education and supervision during antenatal exercises for better postpartum health outcomes [55, 93, 102], and the provision of PA and exercise opportunities in the community setting [55] as interventionist approaches in promoting physical activity during pregnancy. Given the physical and psychological health benefits of regular physical activity, and when juxtaposed with the low prenatal inactivity of pregnant women in Africa as indicated in this review, interventions are needed to enhance the self-efficacy of pregnant women on increasing their physical activity [133]. Providing prenatal physical activity education to pregnant women is one among other intervention strategies to change the physical activity behaviour of women. Efforts to help pregnant women realise their goal in achieving sufficient physical activity are desirable. In this regard, health professionals working with pregnant women, amidst other specialist disciplines such as exercise physiologists, physiotherapists and biokineticists (who practise exclusively in South Africa and Nambia) in the provision of prenatal physical activity education need to be drawn in, and further provision of exercise programmes and social support within the community would also be helpful. Definably, biokineticists are clinical exercise specialists that prescribe individualised exercise and physical activity for rehabilitation and promotion of health and quality of life [134]. The interventions’ components could include one-on-one physical activity during antenatal sessions at the health facilities [135, 136], sharing of information and advice through booklets, leaflets, or websites [135, 136], and a combination of both counselling sessions and information provision without the use of exercise classes [137]. Whatever the outcome of the intervention, it should be tailored to the needs and context of the pregnant woman. There is a scarcity of studies exploring interventions to promote physical activity in Africa; therefore, such studies are needed to guide and promote optimal participation in physical activity during pregnancy among women in Africa.

### Limitations and strengths

The limitations of this review should be considered when interpreting the findings. First, the articles reviewed for inclusion were limited to published studies in the English language; it is possible that publications in other languages were not found due to the databases used. It should be noted that there are variations of findings between studies due to the heterogeneity of the methodology used in assessing physical activity and the reported physical activity outcomes; therefore, we reported the results cautiously and relied on what the authors of the included papers had reported. Specifically, the majority of the studies utilised indirect measures (self-reported) rather than objective measurements of PA; therefore, the issue of bias in the reported results cannot be ruled out. In addition, most of the studies were cross-sectional, and data derived from cross-sectional studies cannot establish PA patterns over time, and make it difficult to ascertain causality of the outcome variables. Longitudinal studies are needed to assess PA patterns of pregnant women during pregnancy in Africa. In this regard, the findings reported in these studies should be interpreted with caution. Nevertheless, the dearth of studies reporting on prenatal PA and health outcomes in African countries, calls for concerted research evaluating physical activity during pregnancy, bearing in mind the different cultural, socio-economic and geographic context of Africa. Such information may help to guide contextually tailored interventions to promote prenatal activity among women in Africa. In addition, based on these limitations, we cannot draw firm conclusions on the specific aims of the review and calls for more robust studies, and advocate for future research using objectively measured physical activity and methodological quality studies, which may help foster better data to inform policy and practice. Notwithstanding these limitations, this narrative review has strengths. To our knowledge, this is the first narrative review to comprehensively assess the dynamics of PA participation during pregnancy in Africa. Finally, this review provides quantitative and qualitative information on the level and correlates of PA participation during pregnancy, including the beliefs, sources of information, perceived benefits, barriers and attitudes of pregnant women concerning PA and exercise participation in Africa. It also considers intervention strategies through descriptive statistics and narrative reporting. Due to the number of studies consulted, and their various geographical settings across Africa, the findings reported in this review paper may be considered comprehensive and reflective of the views of pregnant women in Africa. This review holds the promise of generating well-informed and country-or context-specific PA intervention programmes for pregnancy in Africa. Currently scarce empirical investigations exist on PA during pregnancy in the African context.

## Conclusion and implications

The findings of this narrative review are worrying. Most pregnant women in Africa do not participate in PA during pregnancy, largely due to a lack of knowledge on types of PA and exercise recommended for pregnancy, and lack of knowledge regarding the benefits of PA during pregnancy. Generally, research on the levels and correlates of PA participation during pregnancy is scarce. The global call to prioritise PA participation among the general population naturally necessitates further studies on the actual status of PA among different groups and investigations into the factors that hinder and promote it in various settings. Research into these matters among pregnant women in Africa is particularly important because pregnancy is a stage in a woman’s life that has far-reaching implications for her own life and the child. The possible adverse health outcomes associated with physical inactivity among pregnant women could have long-term effects on the already economically constrained and over-burdened healthcare systems of most African countries.

## Data Availability

All data analysed are included in this article.

## Abbreviations

ACOG: American College of Obstetricians and Gynaecologists
AEE: Activity Energy Expenditure
GPAQ: Global Physical Activity Questionnaire
MVPA: Moderate-to- Vigorous Physical Activity
PA: Physical Activity
PAL: Physical Activity Level
PFM: Pelvic Floor Muscle
PPAQ: Pregnancy Physical Activity Questionnaire
PPWR: Post Partum Weight Retention
RCT: Randomised Controlled Trial
RPE: Borge Rating of Perceived Exertion
WHO: World Health Organization
